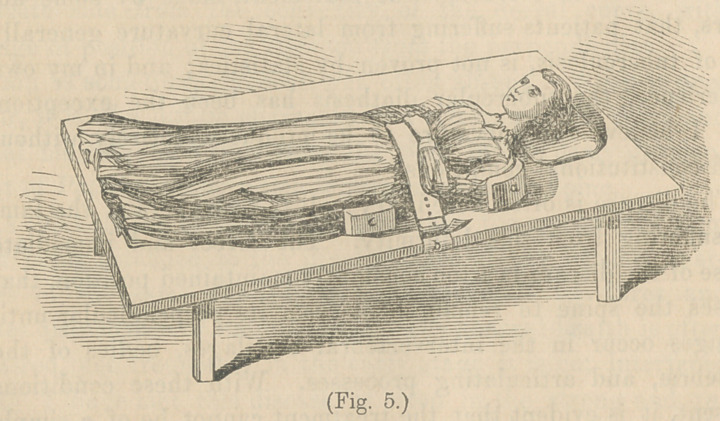# Lateral Curvature of the Spine

**Published:** 1869-02

**Authors:** J. S. Sherman

**Affiliations:** Lecturer on Orthopædic Surgery, in Chicago Medical College; 81 Monroe Street


ARTICLE VII.

### LATERAL CURVATURE OF THE SPINE.

BY J. S. SHERMAN, M.D., Lecturer on Orthopædic Surgery, in Chicago Medical College.

Within the last twenty years, the advance of general surgery has been very rapid; yet, until within the last ten, the subject of deformities has been greatly neglected, and the descriptions of them by surgical writers, considered as authority, are exceedinglyd eficient. From the infrequency of *post-mortem* examinations, the study of spinal curvature has not been generally pursued, and we find many modes of treatment advised, which certainly are not based on the pathological conditions present; and, consequently, the various theories advanced, as to the cause, progress, and treatment of these diseases, are diametrically opposed; facts being taken for granted, without accurate knowledge of the conditions under which these curves must occur.

(Fig. 1.)

The largest number of cases, and most accurate descriptions of *post-mortem* appearances, have been reported by Mr. Adams. His investigations have undoubtedly proven facts heretofore not considered, and I have never seen better results than under his reattment, in the Royal Orthopædic Hospital of London.

The lateral curvature of the spine, as shown by tracing the spinous processes, is usually taken as an index of the curvature. No greater error can occur. A decided internal curvature, involving the bodies of the vertebræ, may exist, with but slight, if any, deviation of the spinous processes.

These curves are all compound, and rotation of the vertebræ on a horizontal plane is a constant factor of all.

The position which they assume, as rotation advances, is shown in Fig. 1, from Adams' Lectures on Spinal Curvatures. The spinous processes remain almost stationary, while the bodies revolve round them, as a centre.

Figs. 2 and 3 are from a case in the Chicago Medical Col-

(Fig. 2.)

lege. (*b*) represents the back view, and shows the curve formed by the spinous processes; (*a*), the front view of the same, formed by the bodies. This is a most aggravated case; yet, the preponderance, to a great degree, of the internal curve over the external is plainly visible, and shows the error of estimating the position of the column by the position of these pro-

(Fig. 3.)

cesses. Serious cases of lateral curvature are often dismissed by the surgeon as of little consequence, because he detects so slight a deviation of the spinous processes from their normal line. These very cases have often reached a degree of internal curvature that produces compression of the viscera, and is slowly changing the form of the bones, to an extent that when external curvature is decided the perfect cure becomes impossible. We must, therefore, look elsewhere than to the spinous processes for early diagnosis.

The investigations of Mr. Adams have thoroughly proven that rotation of the vertebræ is the first step in the abnormal process, and that this rotation is at the expense of the articulating processes, which, in their natural form and position, prevent this movement. Certain symptoms, nevertheless, do indicate curvature in its early stage; and these are: prominence of the scapula and elevation of the shoulder, due to rotary movement, which carries backward the angles of the ribs, on the convex side; and, when the curve is double, a prominence of the hip is seen, produced by the retraction of the abdominal muscles into the concavity of the lumbar curve; also, on the anterior surface of the body, a projection of the breast, on the opposite side from the convexity of the dorsal curve, with change of position of the sternum from the perpendicular.

Pain is sometimes present, yet often absent. When it does occur, it is of a diffuse character, and referred to the spine and spinal muscles; differing from the pain in the early development of Potts' disease, which is remote from the spine.

As the twisting of the column advances, the spinal cord also, from being attached to the inner surface of the foramen, partakes of the same change, which compresses its fibres and causes disturbance of its functions. The general health is often impaired, yet frequently in the early stage it is not interfered with. I have met this disease as often in the strong and robust as in the feeble and weak; but most cases of advanced curvature suffer *some* impairment of health.

When lateral deviation of the spinous processes is seen, it is at the expense of the *cartilages* and *bones;* the latter are only altered in shape in cases of long standing.

When we consider that the sum of the thickness of all the intervertebral cartilages is equal to one-fifth or one-fourth of the height of the spinal column, and that pressure, in the upright position, for twelve hours, diminishes the height of the individual from three-quarters to one inch, by simple compression of these bodies, it is not difficult to account for lateral curvature being produced by continued uneven pressure upon the same. When the extreme point of compression is reached in this tissue, the bones begin to yield; and Fig. 3 shows, also, the wedge shape of both bones and cartilage.

The early recognition of curvatures becomes more important, when we consider that their correction and cure in the early stages is easily accomplished; while in the old and confirmed, the deformity yields exceedingly slow.

The formation of some is laid in infancy; the vertebræ are not completely consolidated until near the thirtieth year, and deficiency of development or irregularity in form are often not noticed in early life, but show their effects when the normal, dorsal, and lumbar curves begin to develop, generally between the ages of eight and seventeen. This class are among the most difficult to correct. The statement, made by some authors, that patients suffering from lateral curvature generally die of tuberculosis, is not proven by statistics; and in my own experience, the tubercular diathesis has been the exception, and I believe the curvature to be produced entirely without such constitutional conditions.

The disease is often hereditary; all the members of the family suffering from this deformity. The direct and immediate cause of the disease is a too constantly maintained position, that causes the spine to remain bent from its perpendicular until changes occur in the intervertebral cartilages, bodies of the vertebræ, and articulating processes. With these conditions present, it is evident that the treatment cannot be of a simple character. Simple exercise of muscles will not correct changes which have occurred in the bones and cartilages; nor will pressure alone, on the convexities of the curves, remove the rotation. A combination of lateral and rotary movement is absolutely necessary.

For the purpose of making lateral pressure in the *up ight* position, I use the ratchet and pivot instrument, figured in No. 4. The lateral pressure is made by means of the steel plates attached to the upright rods at the back, and the force is regulated by turning the ratchets below.

(Fig. 4.)

This instrument has been modified by various orthopædic surgeons, and Mr. Adams combines a rotary movement of the side-plates. I have preferred to make this movement with the patient in a horizontal position, as the weight of the body is then removed and rotation more easily accomplished.

Fig. 5 represents the patient on the spinal couch: (*a*) being a strong pad, curved to fit the shoulder, and (*c*) a similar one,

(Fig. 5.)

adjusted to the hip; (*b*) is a strong *elastic* band, passing round the body. The under portion of this band is attached to a buckle on the side. Its tendency, when tightened, is to compress and, at the same time, twist, or rotate, the ribs in an opposite direction, on the same principle that a band revolves the wheel over which it passes. This is more effectual than any apparatus applied when the patient is standing. Strong lateral force is produced at the same time.

Continuous use of these means is necessary for the correction of spinal deformities. There is no rapid mode of correcting them; weeks and months are necessary. By using the supporter and the lounge, the patient is able to get all the exercise necessary for health; and, in severe cases, the lounge should be used at least six or eight hours out of the twenty-four.

*81 Monroe Street.*

## Figures and Tables

**Fig. 1. f1:**
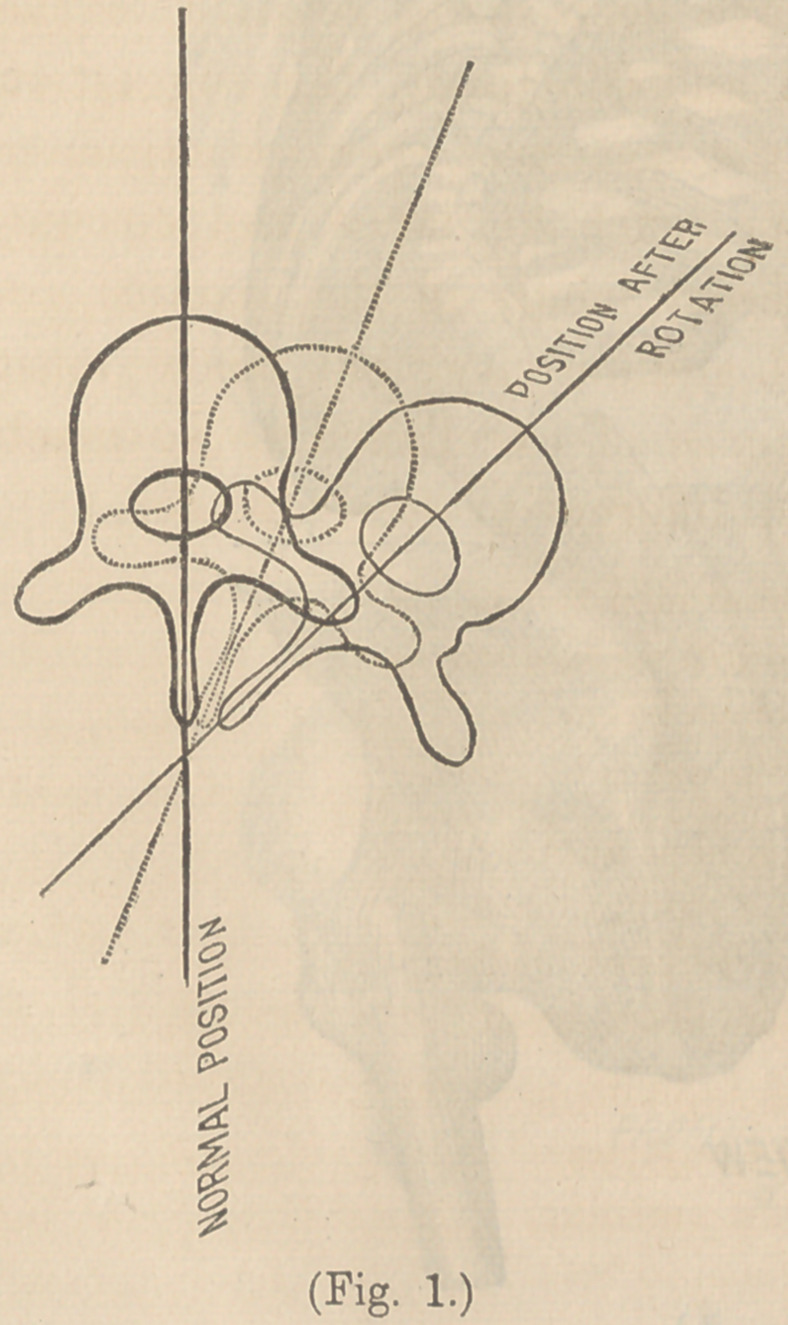


**Fig. 2. f2:**
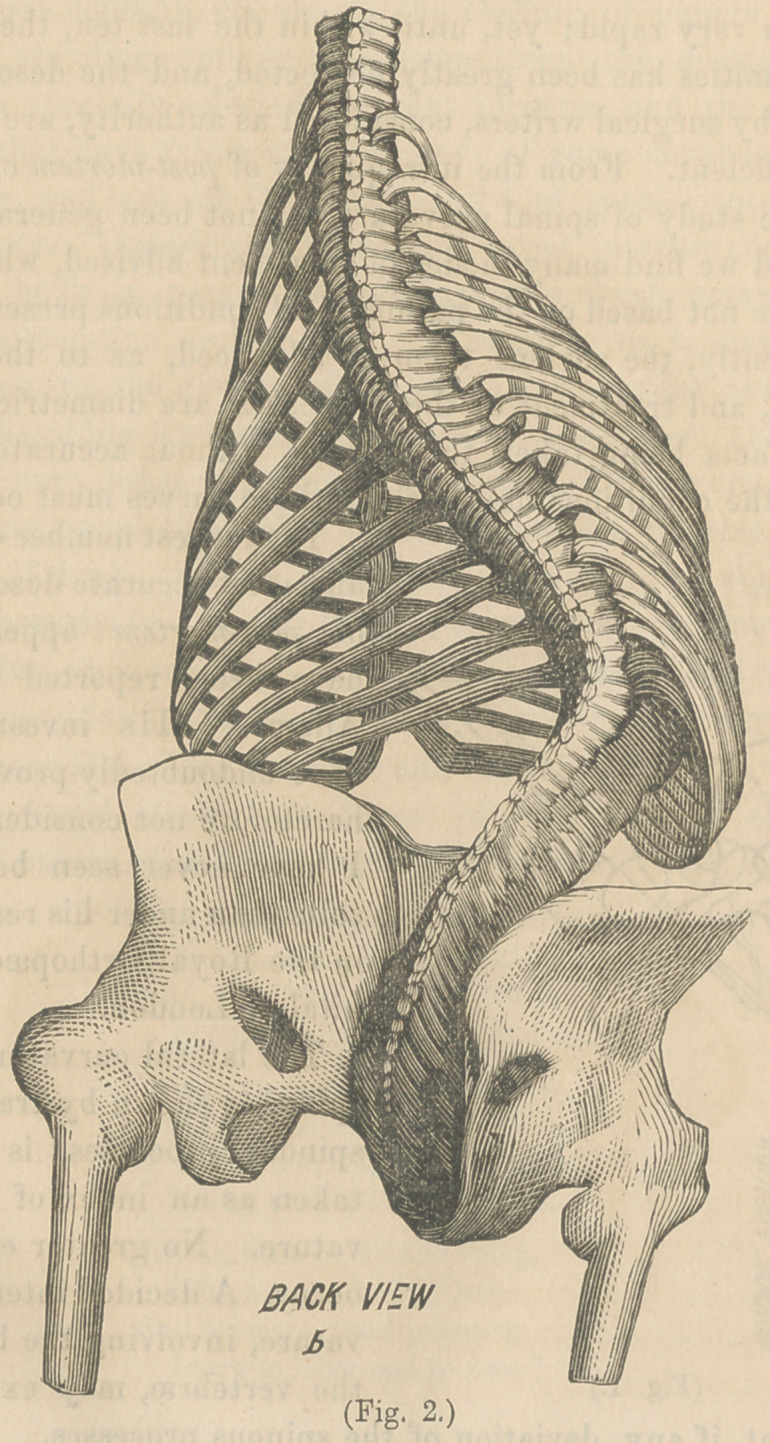


**Fig. 3. f3:**
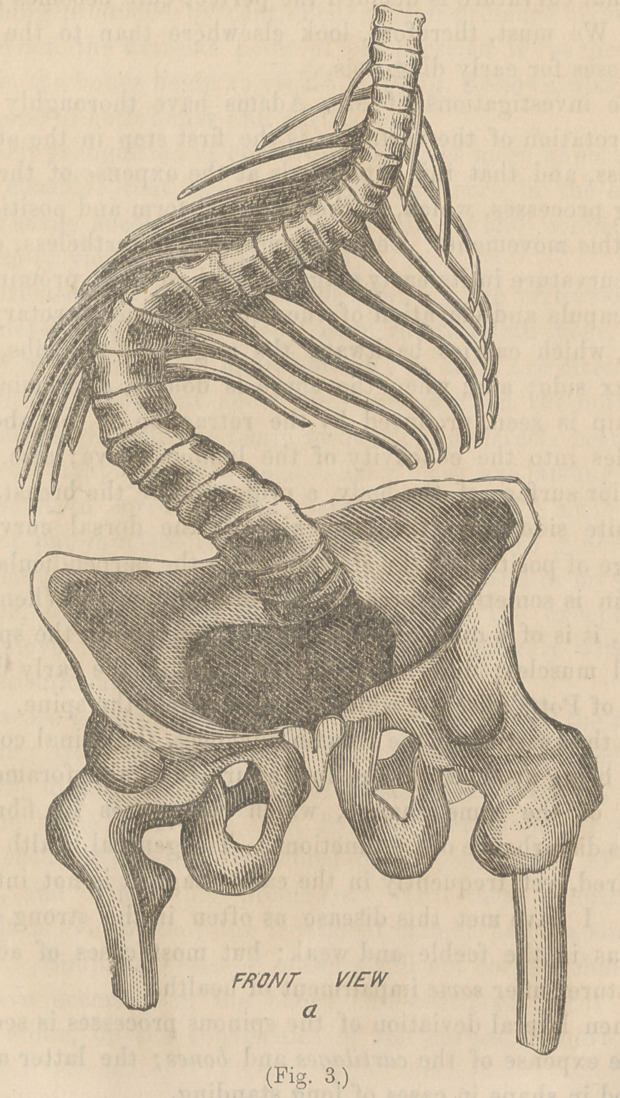


**Fig. 4. f4:**
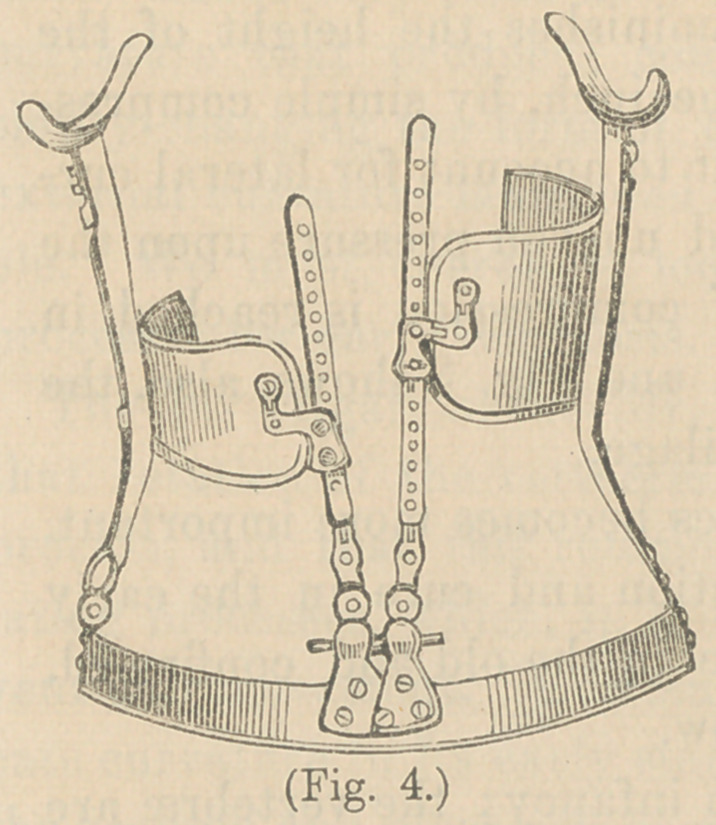


**Fig. 5. f5:**